# Molecular Epidemiology of Blood-Borne Human Parasites in a *Loa loa*-, *Mansonella perstans*-, and *Plasmodium falciparum*-Endemic Region of Cameroon

**DOI:** 10.4269/ajtmh.15-0746

**Published:** 2016-06-01

**Authors:** Papa M. Drame, Céline Montavon, Sébastien D. Pion, Joseph Kubofcik, Michael P. Fay, Thomas B. Nutman

**Affiliations:** Helminth Immunology Section, Laboratory of Parasitic Diseases, National Institute of Allergy and Infectious Diseases, National Institutes of Health, Bethesda, Maryland; Institut de Recherche pour le Développement, Montpellier, France; University of Montpellier, Montpellier, France; Biostatistics Research Branch, National Institute of Allergy and Infectious Diseases, National Institutes of Health, Bethesda, Maryland

## Abstract

The study of the interactions among parasites within their hosts is crucial to the understanding of epidemiology of disease and for the design of effective control strategies. We have conducted an assessment of infections with *Loa loa*, *Mansonella perstans*, *Wuchereria bancrofti*, and *Plasmodium falciparum* in eastern Cameroon using a highly sensitive and specific quantitative polymerase chain reaction assay using archived dried whole blood spots. The resident population (*N* = 1,085) was parasitized with *M. perstans* (76%), *L. loa* (39%), and *P. falciparum* (33%), but not with *W. bancrofti*. Compared with single infections (40.1%), coinfection was more common (48.8%): 21.0% had *L. loa*–*M. perstans* (Ll^+^/Mp^+^/Pf^−^), 2.7% had *L. loa*–*P. falciparum* (Ll^+^/Pf^+^/Mp^−^), 15.1% had *M. perstans*–*P. falciparum* (Mp^+^/Pf^+^/Ll^−^), and 10.0% had *L. loa*–*M. perstans*–*P. falciparum* (Ll^+^/Mp^+^/Pf^+^). Interestingly, those with all three infections (Ll^+^/Mp^+^/Pf^+^) had significantly higher *L. loa* microfilaria (mf) counts than either single Ll^+^ (*P* = 0.004) or double Ll^+^/Mp^+^ (*P* = 0.024) infected individuals. Of those infected with *L. loa*, the mean estimated counts of *L. loa* mf varied based on location and were positively correlated with estimated intensities of *M. perstans* mf. Finally, at a community level, heavy *L. loa* infections were concentrated in a few individuals whereby they were likely the major reservoir for infection.

## Background

Humans are subject to infection by a variety of parasites (protozoan and metazoan) throughout their life. In tropical regions, multiple parasitic infections are the rule, given their overlapping endemicity.^1^ Helminth coinfections alone affect over 800 million people and outnumber single (or no) infection in many communities living in low-income, resource-limited parts of the world.^2^–^6^

Although soil-transmitted helminths (hookworms, *Ascaris*, and *Trichuris*) represent the greatest proportion of coinfecting species,^7^–^10^ blood-borne infections involving filarial and malarial parasites have been described in many parts of the world. For example, coinfections with *Plasmodium* spp. and *Wuchereria bancrofti*, which can be transmitted by the same vectors, have been described in India,^11^ Guyana,^12^ Kenya,^13^ Mali,^14^ and Tanzania^4^ among many other countries. Dual infections with *Plasmodium* spp. and either *Loa loa*, *Mansonella perstans* or *Mansonella ozzardi*, three clinically less-important filarial parasites, have been seen in populations living in Uganda, Cameroon, and Argentina.^10^,^15^–^17^ Coinfections involving only filarial helminths are similarly well known: *L. loa* and *M. perstans* dual infections have been observed in Cameroon,^18^ Congo,^19^ Gabon,^20^,^21^ and Nigeria,^22^ and *L. loa*–*Onchocerca volvulus* coinfection has been described in Cameroon.^23^,^24^ Concomitant infections with three (or more) human filarial helminths have also been reported.^25^

Coinfecting parasites within hosts have been postulated to compete with each other directly for host resources^26^ or indirectly through the host immune system.^27^ Such interactions can alter disease severity, transmission, or infection dynamics. They can then strongly influence the epidemiology of associated diseases.^2^,^28^ For example, in central Africa, treatment of *O. volvulus* (or *W. bancrofti*) in the presence of *L. loa* can cause severe posttreatment side effects that can lead to death.^23^,^29^ *Plasmodium* and filarial helminth coinfections have also been described,^9^,^30^,^31^ with contrasting effects on the epidemiology of malaria, making the nature of such interactions not always predictable. Therefore, the knowledge of interactions among parasites is important in understanding the epidemiology of infections and the diseases they cause.

Recent advances in molecular biology have allowed the development of a range of new, relatively cheap, and rapid high-throughput molecular assays. These molecular tools can be used to examine and quantitate parasites (or their DNA) directly from archived clinical or environmental samples, and then to study the dynamics of specific parasitic infections among host populations.^32^,^33^ These molecular tools offer insights into our understanding of the transmission, pathogenesis, progression, and control of parasitic diseases in situations of polyparasitism.^33^ To date, disease control programs mostly adopt a vertical approach to intervention, dealing with each pathogen in isolation. Because pathogen interactions worsen human health and increase transmission potential, control measures need to be more integrated.

The objective of the study was to determine if a molecular epidemiologic approach could help to understand the population biology of important vector-borne parasitic infections in a previously unmapped region of the world, using anonymized, archived samples that would have been discarded. This was a “proof of principle” study to assess the feasibility of using such an approach with an emphasis on *L. loa* infection in Cameroon, as it has recently gained prominence because of the possible serious adverse events occurring after ivermectin administration. In so doing, we identified epidemiological (demographic and biological) factors that may play a crucial role in the human susceptibility to and the transmission of *L. loa*.

## Methods

### Ethics statement.

This study was derived from a cross-sectional epidemiologic study of human immunodeficiency virus (HIV) and syphilis prevalence. This study received ethical clearance from the National Ethics Committee of Cameroon. The objectives of the study were explained orally to all eligible individuals. Written informed consent was obtained from all participants or from an adult parent or guardian for children. This study used anonymized and coded individual samples that were about to be discarded. These were deemed exempted from National Institutes of Health Institutional Review Board approvals by the Office of Human Subjects Research.

### Study area.

The study was conducted in the district of Messok (3°05′04.9″N and 14°03′48.9″E), a rural area located in the Haut-Nyong Department, in the eastern region of Cameroon. It is a densely forested area with an annual average rainfall of 2,800 mm, the rainy season lasting from March to November. Average temperatures monthly varied between 22°C and 25°C. This region had not been previously mapped for parasitic infections. All individuals from eight villages—Bareko, Karagoua, Kamelone, Long, Messok, Messea, Nkouakom, and Yanebot—were invited to participate. Messok village was the largest and the focal point village. There had been no mass drug administration programs for control of onchocerciasis, lymphatic filariasis, or soil-transmitted helminths.

### Sample collection.

All samples were archived samples that had been acquired as part of a cross-sectional survey for sexually transmitted infections, namely HIV and syphilis, between October and November 2012. As mentioned above, these were coded, anonymized convenience samples from healthy volunteering individuals aged ≥ 2 years. Whole blood had been collected by venipuncture during the day from 10:00 am to 4:00 pm, and 50 μL were spotted onto filter paper (Whatman no. 1 filter paper). Blood spots were then sun/air-dried for 15–20 minutes and stored at room temperature until used for DNA extraction. Each dried blood-spotted filter paper was kept individually in a paper pouch in such a way that samples from each individual were physically separated from one another.

### DNA extraction for field-collected blood spot samples.

DNA was extracted from the dried blood spots as previously described.^34^ In brief, two punched (4 mm each) blood spots were immersed in 400 μL water in a tube containing ceramic beads (Peqlab, Wilmington, DE). These tubes were then homogenized at 6,500 × *g* for 5 minutes, and then heated for 30 minutes at 99°C while shaking in a thermomixer (Eppendorf North America, New York, NY). Finally, the samples were centrifuged at 15,700 × *g* for 10 minutes, and the supernatant was collected for use.

### Extraction of genomic DNA.

Genomic DNA (used as positive controls) was obtained from microfilariae of *L. loa*, *M. perstans*, and *W. bancrofti* and from *P. falciparum* parasites using a previously described method.^35^

### Quantitative polymerase chain reaction assays.

Primers and probes for specific amplification of *L. loa* microfilaria (mf), *M. perstans* mf, *W. bancrofti* mf, and *P. falciparum* (GenBank accession nos. HM753552.1, U31640.1, U31644.1, and NC_004331, respectively) have been described previously^35^–^37^ and are listed in Supplemental Table 1.

Quantitative polymerase chain reaction (qPCR) assays for detection of genomic DNA of *L. loa* mf, *M. perstans* mf, and *W. bancrofti* mf and *P. falciparum* were performed on a Viia7 detection system (Life Technologies, Grand Island, NY) using TaqMan Kapa Probe Fast qPCR Kit reagents (Kapa Biosystems Inc., Wilmington, MA) and primer/probe sets described in Supplemental Table 1. All assays were done in a 10-μL reaction mixture consisting of 5 μL of 2 × KAPA (Kapa Biosystems Inc.) buffer, 0.9 ηM of each primer and 0.25 ηM of the corresponding 6-carboxyfluorescein (FAM) probe, 2 μL of extracted DNA (template), and 2.75 μL ultrapure water. Genomic DNAs were used as positive controls, ultrapure water as negative controls (no template controls [NTCs]), and all assays were run in duplicate. Amplification conditions were 20 seconds at 95°C followed by 40 cycles of 1 second at 95°C and 20 seconds at 60°C. The quality of templates was confirmed by testing all samples for the ability to amplify a control plasmid DNA (New England BioLabs Inc., Ipswich, MA) added before DNA extraction to ensure a lack of inhibition.

### Assessment of parasitic infections.

A sample was considered positive if the read cycle number (Ct) value was at least two Ct values lower than the mean value of the NTCs (Ct_NTCs_). The estimated number of *L. loa* mf was obtained by extrapolating from a standard curve derived from blood samples spiked with dilutions of a known count of mf as previously described.^35^ *Mansonella perstans* mf, *W. bancrofti* mf, and *P. falciparum* parasites were quantified using a relative method based on the following formula: 2^−(ΔCt)^, where ΔCt = Ct_x_ (mean duplicates of sample x) − Ct_NTCs_ (mean values of negative controls [water]).

### Statistical analyses.

**Figure 1.**
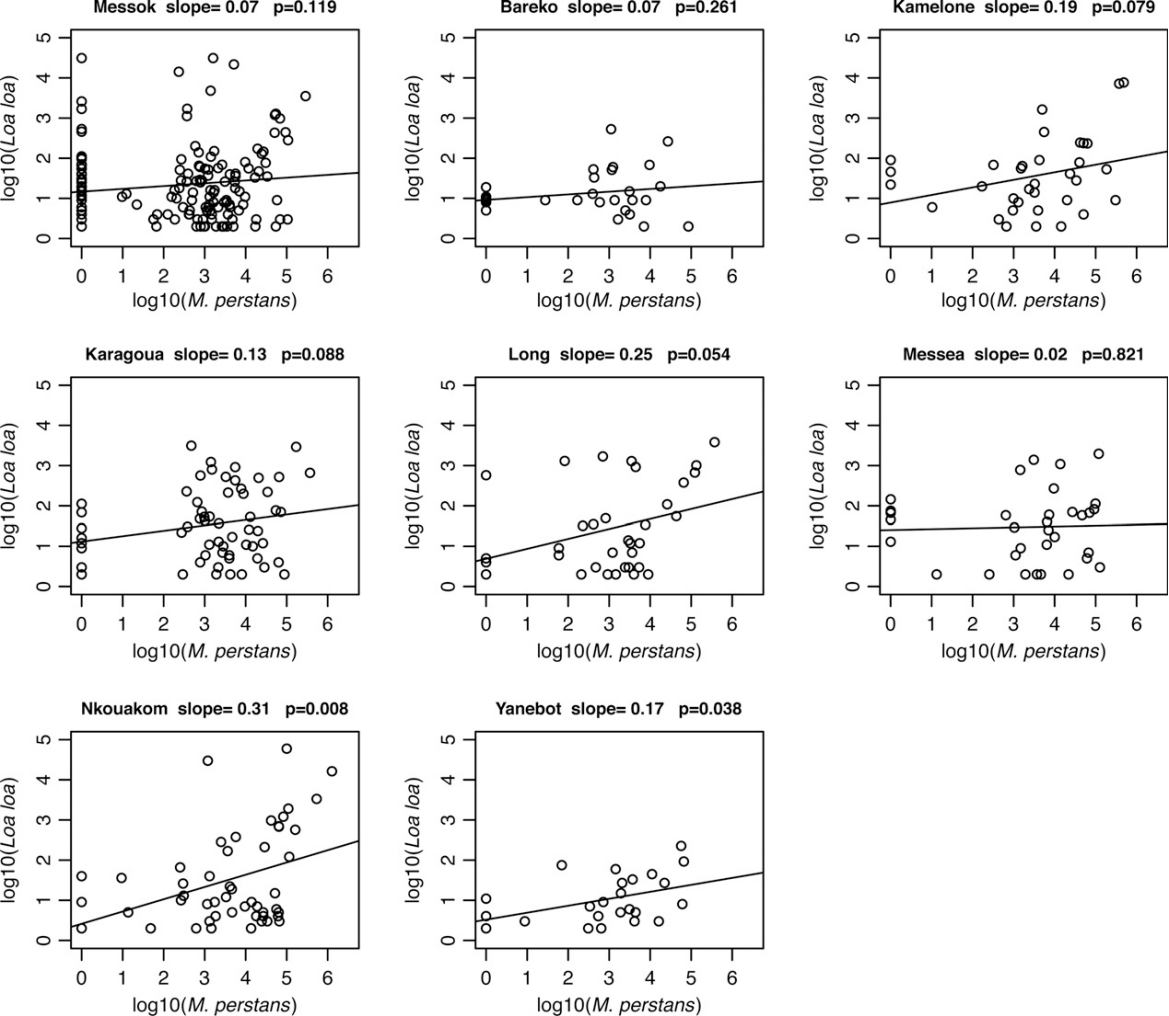
Association between *Loa loa* and *Mansonella perstans* infections based on the location of residence. A separate linear model was fit for each village to evaluate the strength of the association between *L. loa* microfilaria loads and the relative intensities of *M. perstans* infection in each of the eight villages studied. The slope of the line and the *P* value of significance are indicated for each village.

Fisher's exact test was used to compare parasite prevalence between groups (when there were more than two groups, the Fisher's exact test was calculated by simulating with 100,000 replications), and the nonparametric Mann–Whitney (two groups) tests were used to estimate difference in parasite intensities. We used a hurdle method to model *L. loa* counts. This is a two-part model. The first part is a logistic regression that models *L. loa* presence or absence and the second part models the positive *L. loa* counts using a zero-truncated quasi-Poisson model. The regressors were the same for both parts of the model: host age (eight categories), gender, location (eight villages), and log_10_ intensity (undetectable counts are set to 1, so that their log_10_ values are 0) of coinfection with *M. perstans* or *P. falciparum*. For statistical inferences, we used the Wald method for the logistic model and nonparametric bootstrap method (*B* = 2,000 replications, percentile confidence intervals) for the truncated quasi-Poisson model. The latter method is used for the truncated model because it is more robust to model misspecification. No tests were done on the village or age effects for the truncated model because it is not straightforward to apply the bootstrap in those cases. The slopes and *P* values in Figure 1

are linear models within each village. Analyses were done using R software version 3.2.0 and the hurdle models used the pscl R package.^38^

## Results

A total of 1,170 samples were tested for *L. loa*, *W. bancrofti*, and *M. perstans* and for *P. falciparum* detection and quantification. Of these, 26 samples were not interpretable because the positive control for DNA extraction (spiked pBR322 plasmid) was unamplifiable. The final data set thus had only *N* = 1,085 subjects, as an additional 59 subjects had missing gender information, and 58 of those 59 had missing age information as well.

### General characteristics.

The resulting studied population (*N* = 1,085) included 613 women (56.5%) and 472 men (43.5%) with an age distribution shown in Table 1. The gender ratio did not differ by location (*P* = 0.94) but the age distribution did differ among the villages (*P* < 0.001).

### Prevalence of parasite species.

In the analyzed population (*N* = 1,085), the prevalence of infection with *M. perstans* mf, *L. loa* mf, and *P. falciparum* was 75.9%, 38.7%, and 33.3%, respectively. No *W. bancrofti* infection was detected. The prevalence ranged between 27.6% and 62.0% for *L. loa*, 64.4% and 89.1% for *M. perstans*, and 23.1% and 37.0% for *P. falciparum* (Table 2). For each parasite studied, the prevalence of infection was higher in men compared with women: 80.3% versus 72.4% for *M. perstans* (*P* = 0.003), 42.6% versus 35.7% for *L. loa* (*P* = 0.02), and 36.9% versus 30.5% for *P. falciparum* (*P* = 0.03). We found significant age effects for *P. falciparum* prevalence (*P* < 0.001) and *M. perstans* prevalence (*P* = 0.003), but not for *L. loa* mf prevalence (*P* = 0.97).

**Figure 2.**
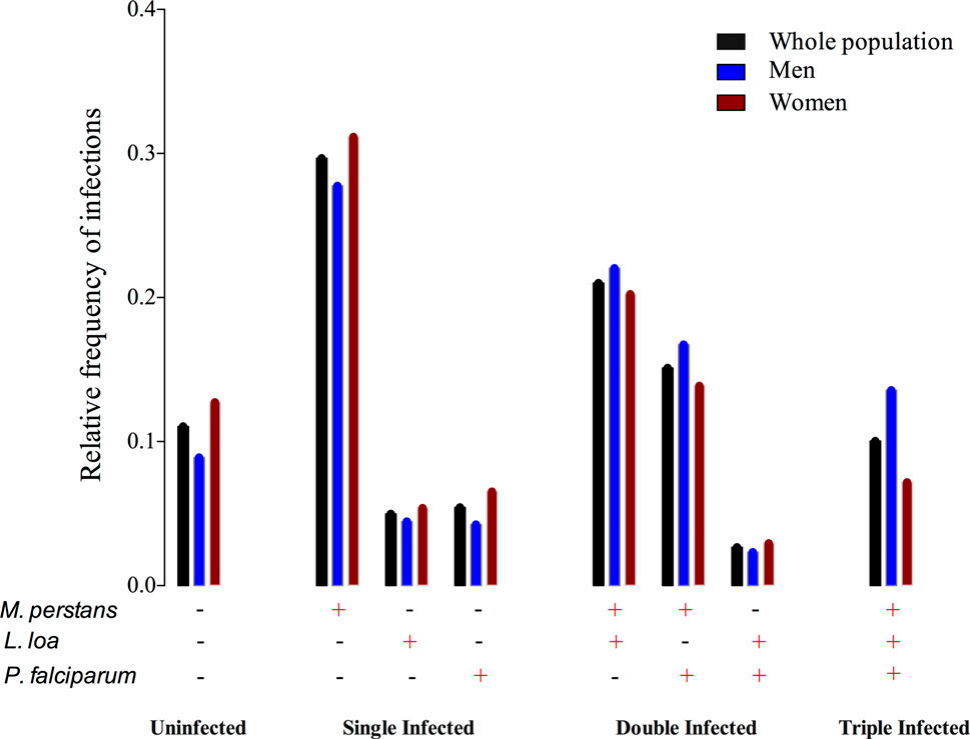
Distribution of species prevalences within the host population. The relative frequency of single, double, and triple infections with *Loa loa*, *Mansonella perstans*, and/or *Plasmodium falciparum* are represented for the whole population (black), for men (blue), and for women (dark red). The “+” and “−” signs indicate the presence (+) or absence (−) of the corresponding species.

The distribution of infection combinations is shown in Figure 2

. Only 11.1% of individuals were completely uninfected with any of the parasite species tested. Thus, 88.9% had at least one infection: 40.1% had single infections and 48.8% had two or more infections. In detail, 21.0% were *L. loa* mf–*M. perstans* mf coinfected, 2.7% were *L. loa* mf–*P. falciparum* positive, 15.1% had *M. perstans* mf–*P. falciparum* infections, and 10.0% were infected with all three species (Figure 2).

### Factors influencing susceptibility to and intensity of *L. loa* infection.

**Figure 3.**
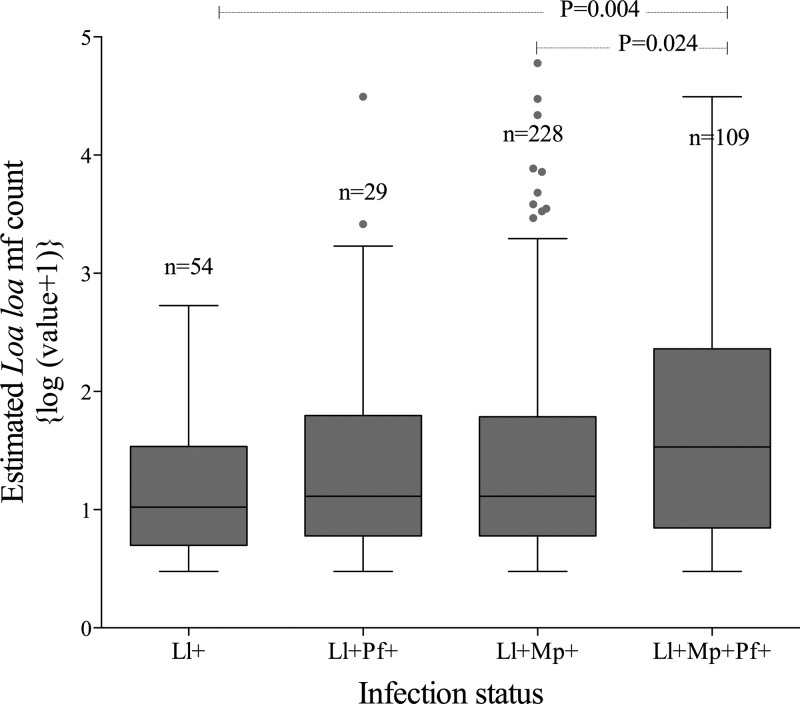
Level of *Loa loa* microfilaria (mf) counts according to the infection status. A comparison of the estimated counts of *L. loa* mf/mL among those with single *L. loa* (Ll^+^), double *L. loa*/*Mansonella perstans* (Ll^+^/Mp^+^) and *L. loa*/*Plasmodium falciparum* (Ll^+^/Pf^+^), and triple *L. loa*/*M. perstans*/*P. falciparum* (Ll^+^/Mp^+^/Pf^+^) infections.

Taking *L. loa* infection as the infection of interest, people with triple infections (Ll^+^/Mp^+^/Pf^+^) had higher estimated *L. loa* mf counts than either single *L. loa* (Ll^+^; *P* = 0.004) or double *L. loa*–*M. perstans* (Ll^+^/Mp^+^; *P* = 0.024). The estimated *L. loa* mf counts in individuals with triple infection were not significantly higher than *L. loa* mf–*P. falciparum* (Ll^+^/Pf^+^; *P* = 0.25) infected individuals (Figure 3

).

On the basis of a logistic model of *L. loa* mf prevalence (part 1 of the hurdle model), village location had a highly significant effect (*P* < 0.001) but age (*P* = 0.86) and gender (*P* = 0.09) played little to no role. The logistic model shows that each log_10_ increase in *M. perstans* intensity increases the odds of *L. loa* presence by a factor of 1.23 (95% confidence interval [CI] = 1.13–1.34; *P* < 0.001), while there is no analogous change in odds for each log_10_ increase in *P. falciparum* intensity (odds ratio [OR] = 0.93, 95% CI = 0.81–1.07, *P* = 0.30).

On the basis of the quasi-Poisson model, estimated *L. loa* intensity in males is not significantly different from females (*P* = 0.94). Estimated *L. loa* mf density was positively associated with estimated *M. perstans* mf density, but this association was just at the level of significance (1 log_10_ increase in *M. perstans* mf density was associated with a 1.55-fold increase in *L. loa* mf density; 95% CI = 1.00–4.04, *P* = 0.051). Estimated *P. falciparum* intensity had no effect on estimated *L. loa* mf density (1 log_10_ increase in *P. falciparum* density was associated with only a 1.06-fold increase in *L. loa* mf density; 95% CI = 0.52–2.01, *P* = 0.80).

### Association between *L. loa* and *M. perstans* infections based on location.

In Figure 1, we represented the estimated *L. loa* mf density as a function of estimated *M. perstans* intensity for each village for those with *L. loa* infection. We fitted linear models and tested that the slope is different from zero. Although we only see significantly positive slopes (positive association between *L. loa* and *M. perstans* densities) in two villages (Nkouakom, *P* = 0.008 and Yanebot, *P* = 0.038), all of the eight villages estimate had positive slopes. Thus, this analysis also supports a positive association between *L. loa* and *M. perstans*.

### Community distribution of *L. loa* and *M. perstans* microfilarial densities.

**Figure 4.**
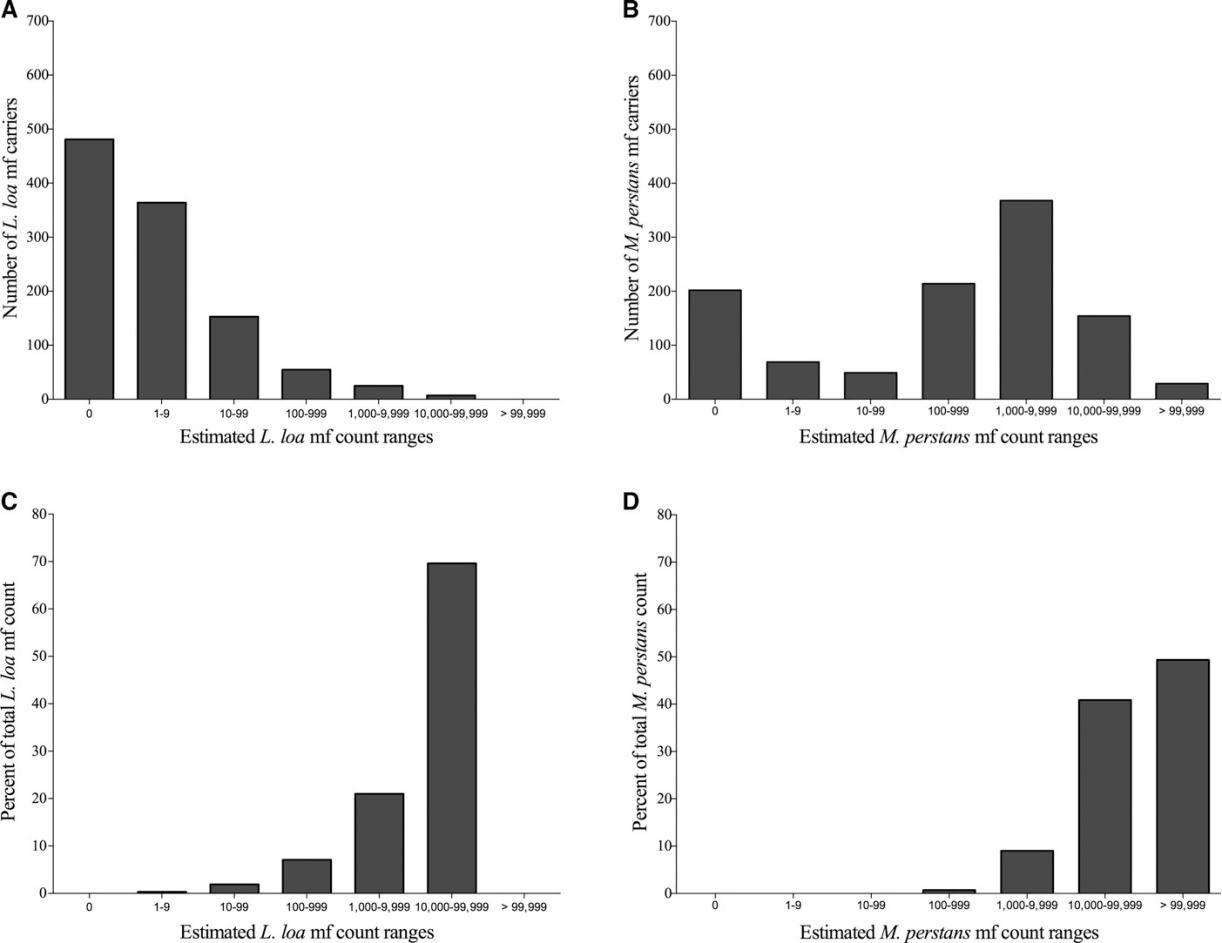
Community distribution of *Loa loa* and *Mansonella perstans*. (**A**) The number of individuals with a given range of *L. loa* or (**B**) *M. perstans* are shown. The contribution of each of these ranges to the community microfilarial load (total of community mf) for *L. loa* mf (**C**) or *M. perstans* mf (**D**) is shown as a percent of the total community mf count.

Because estimated *L. loa* and *M. perstans* mf loads appeared to be strongly associated, we were interested to see how these two parasites were distributed within the studied communities. A more detailed analysis shows that 0.6% (7/1,085) of people harbored more than 10,000 *L. loa* mf/mL of blood and contributed to more than 69.7% of the total estimated *L. loa* mf load in the studied communities and that 2.9% (32/1,085) of the population harbored 90.7% of the estimated community mf loads (Figure 4A and C

). Of note, three individuals (one man and two women) were parasitized with more than 30,000 *L. loa* mf/mL of blood. In contrast, for *M. perstans*, it required 183 (16.9% of people) most heavily infected individuals to contribute to 90.2% of the estimated community mf load (Figure 4B and C).

## Discussion

The knowledge of the interactions among parasites within their hosts is crucial to the understanding of transmission, pathogenesis, patterns of disease, and for the design of effective control and elimination strategies.^33^ This study demonstrates the utility of molecular tools to drive a post hoc epidemiological assessment of the prevalences and intensities of (co)infections/transmission of three blood-borne filarial and one malaria parasites (*L. loa*, *W. bancrofti*, *M. perstans*, and *P. falciparum*). As *L. loa* mf infection was our main interest in this study, we highlighted as well the epidemiological factors associated with variations of the intensity of *L. loa* mf infection, and likely the transmission of loiasis.

Populations residing in this region of east Cameroon appear to be highly parasitized with *M. perstans* (76%), *L. loa* (39%), and *P. falciparum* (33%). These results confirm previous reports indicating that almost all subjects in endemic regions may be carriers of *M. perstans* mf, whereas the prevalence rate of *L. loa* mf carriers rarely exceeds 40%.^19^,^39^ The absence of *L. loa* mf in a high proportion of the population (61% in this study) may be due to genetically controlled host resistance^40^,^41^ or perhaps limits on exposure.

*Wuchereria bancrofti* was not detected in any sample. We are not aware of any previous epidemiological assessment of lymphatic filariasis in this region. None of our samples was positive for *W. bancrofti* DNA. This probably reflects the true absence of this infection, but it may also be related to the timing of the blood draw in that *W. bancrofti* mf are very low or absent in the peripheral blood during daytime. This being said, given the number of individuals sampled, if *W. bancrofti* were highly endemic, we would have expected to see a number (albeit few) of positives by PCR even during daytime blood draws.^42^

To evaluate species prevalence, we specifically targeted mf of *L. loa*, *M. perstans*, and *W. bancrofti* for detection. Despite being more sensitive than the widely used thick blood smear to detect microfilariae, qPCR-based assays are still likely to underestimate the absolute prevalence of the *L. loa* and *M. perstans* infections given that some individuals with adult parasites are amicrofilaremic. Nevertheless, our data clearly indicate that this region should be considered to be a hyperendemic zone for *L. loa*, *M. perstans*, and *P. falciparum*.

In agreement with previous reports,^43^,^44^ we have shown that multiple infections outnumber single or no infection in the studied region. We have also shown that the intensity of *M. perstans* mf was a significant predictor of the intensity of *L. loa* mf load, but that *P. falciparum* (asexual malaria here) intensity was not, perhaps indicating a coadaptation/coevolution of *L. loa* and *M. perstans* to the host environment.

We have shown that the intensity of *L. loa* mf and the strength of its association with the density of *M. perstans* mf may vary based on geographical location, which is most likely due to a difference in exposure to the vectors that transmit these infections. Thus, the host exposure level promotes the coexistence of these two species. These results point to a mechanism of competition between *L. loa* and *M. perstans*, which can determine the abundance of their microfilariae within a host population.^41^,^43^,^44^ However, we cannot exclude an effect of HIV infection on that association. Indeed, a recent study has reported a higher prevalence of *L. loa* mf in HIV patients in Gabon,^45^ though no comparison was made between HIV-positive and HIV-negative subjects in terms of *L. loa* mf in this study. Although this particular association (HIV status and mf number) would be interesting to study, we were not able to individualize *L. loa* mf counts and HIV status. However, the global prevalence of the studied population ranged up to 7% (E. Delaporte and others, unpublished data), slightly higher than the national prevalence rate in Cameroon, 5.5%.

Interestingly, the fact that few (0.6%) individuals harbor the overwhelming majority (69.7%) of *L. loa* mf suggests that if one had the ability to target these few people with safe and effective drugs, a significant reduction in community microfilarial load could be achieved such that transmission could not be sustained.^23^,^43^ In addition, those highly parasitized *L. loa* microfilaremic individuals are those at high risk for the development post-ivermectin serious adverse events.^23^,^24^

In summary, molecular epidemiologic assessments using tools applicable to previously collected but well-characterized material can provide very important insights into heretofore unexplored coinfections among disparate microorganisms. Such insights would ultimately benefit global health programs generally and provide support to elimination programs for neglected tropical and other infectious diseases.

## Supplementary Material

Supplemental Table.

## Figures and Tables

**Table 1:** General characteristics of the studied population

	Total population (*N* = 1,085)	Bareko (*N* = 50)	Kamelone (*N* = 104)	Karagoua (*N* = 119)	Long (*N* = 118)	Messea (*N* = 113)	Messok (*N* = 402)	Nkouakom (*N* = 92)	Yanebot (*N* = 87)
Gender: % (*n*)									
Male	43.5 (472)	38.0 (19)	41.3 (43)	47.1 (56)	40.7 (48)	42.5 (48)	44.0 (177)	46.7 (43)	43.7 (38)
Female	56.5 (613)	62.0 (31)	58.7 (61)	52.9 (63)	59.3 (70)	57.5 (65)	56.0 (225)	53.3 (49)	56.3 (49)
Age classes (years): % (*n*)									
< 15 (*N* = 90)	8 (90)	4 (2)	–	8 (9)	–	4 (4)	17 (70)	–	6 (5)
15–19 (*N* = 151)	14 (151)	6 (3)	8 (8)	11 (13)	7 (8)	17 (19)	19 (75)	14 (13)	14 (12)
20–29 (*N* = 247)	23 (247)	18 (9)	17 (18)	34 (40)	29 (34)	26 (29)	16 (63)	32 (29)	29 (25)
30–39 (*N* = 196)	18 (195)	20 (10)	22 (23)	28 (33)	19 (22)	12 (14)	15 (62)	21 (19)	14 (12)
40–49 (*N* = 152)	14 (152)	18 (9)	22 (23)	9 (11)	19 (22)	13 (15)	10 (42)	12 (11)	22 (19)
50–59 (*N* = 99)	9 (99)	10 (5)	12 (12)	6 (7)	9 (11)	11 (12)	10 (39)	9 (8)	6 (5)
60–69 (*N* = 62)	6 (62)	2 (1)	10 (10)	2 (2)	8 (9)	4 (5)	6 (25)	9 (8)	2 (2)
> 69 (*N* = 89)	8 (89)	22 (11)	10 (10)	3 (4)	10 (12)	13 (15)	6 (26)	4 (4)	8 (7)

**Table 2:** Prevalences of species according to location

Villages (effective)	*Loa loa* mf % (*n*)	*Mansonella perstans* mf % (*n*)	*Plasmodium falciparum* % (*n*)
Bareko (*N* = 50)	62.0 (31)	74.0 (37)	30.0 (15)
Kamelone (*N* = 104)	32.7 (34)	83.6 (87)	23.1 (24)
Karagoua (*N* = 119)	50.4 (60)	79.8 (95)	34.4 (41)
Long (*N* = 118)	28.8 (34)	83.9 (99)	33.1 (39)
Messea (*N* = 113)	29.2 (33)	81.4 (92)	30.1 (34)
Messok (*N* = 402)	38.3 (154)	64.4 (259)	35.6 (143)
Nkouakom (*N* = 92)	54.3 (50)	89.1 (82)	37.0 (34)
Yanebot (*N* = 87)	27.6 (24)	82.8 (72)	35.6 (31)

mf = microfilaria. Species prevalences are represented as %, and the number of positives is indicated in brackets.
