# Supplementary material for: Molecular Epidemiology of Blood-Borne Human Parasites in a Loa loa-, Mansonella perstans-, and Plasmodium falciparum-Endemic Region of Cameroon
Source: Am J Trop Med Hyg. 2016 Jun 1;94(6):1301–8. doi: 10.4269/ajtmh.15-0746 (PMC4889748; doi:10.4269/ajtmh.15-0746)
Supplement: Supplementary file 1 [file SD5.pdf]

Supplemental Table 1

Sequences of qPCR primers/probes

Species	Target	Sequence	Length (no of nucleotides)
Loa loa	LLMF72 frwd primer	5′-CGGAAGACTCAACGTCAGAAATCA-3′	24
	LLMF72 rev primer	5′-AGGAACGCTTGATGGTGATGT-3′	21
	LLMF72 probe	5′-FAM-CCAACAGCCTGCTTTT-NFQ-3′	16
Mansonella perstans	MPITS frwd primer	5′-CGTTGATATTTGAATGCACGACAATG-3′	26
	MPITS rev primer	5′-GGCCGAAACATTCAATTACCTCAA-3′	24
	MPITS probe	5′-FAM-CCGACGTGCACACCAT-NFQ-3′	16
Wuchereria bancrofti	WBITS frwd primer	5′-TGCACAACAACTATATGGGAATGGT-3′	25
	WBITS rev primer	5′-CCTAAACTCTGGGCCGAAACATT-3′	23
	WBITS probe	5′-FAM-AAACCGGTGATCCTACCTGC-NFQ-3′	20
Plasmodium falciparum	Pfal frwd primer	5′-CTTTTGAGAGGTTTTGTTACTTTGAGTAA-3′	29
	Pfal rev primer	5′-TATTCCATGCTGTAGTATTCAAACACAA-3′	28
	Pfal probe	5′-FAM-TGTTCATAACAGACGGGTAGTCATGATTGAGTT-CAMGBNFQ-3′	23
pBR plasmid	pBR322 frwd primer	5′-CTGGCATTGACCCTGAGTGA-3′	20
	pBR322 rev primer	5′-CACGATACGGGTTACTGATGATGA-3′	24
	pBR322 probe	5′-FAM-CGCCGCATCCATACCGCCAG-NFQ-3′	20

FAM = 6-carboxyfluorescein; frwd = forward; MPITS and WBITS = *M. perstans* and *W. bancrofti* mf 5S ribosomal RNA intragenic spacer region, respectively; Pfal = *P. falciparum*; qPCR = quantitative polymerase chain reaction; rev = reverse.
